# Case Report: A novel mixture of dose-fractioned radiation and immunotherapy for treatment of cholangiocarcinoma

**DOI:** 10.3389/fimmu.2023.1273962

**Published:** 2023-12-12

**Authors:** Ningyu Wang, Linfang Wang, Ai Huang, Jun Han, Ting Cao, Xiangping Mei, Jing Yao, Yong Xiao, Hong Ma

**Affiliations:** ^1^Cancer Center, Union Hospital, Tongji Medical College, Huazhong University of Science and Technology, Wuhan, China; ^2^Institute of Radiation Oncology, Union Hospital, Tongji Medical College, Huazhong University of Science and Technology, Wuhan, China; ^3^Department of Gastrointestinal Surgery, Union Hospital, Tongji Medical College, Huazhong University of Science and Technology, Wuhan, China; ^4^Department of Radiation Oncology, Hubei Cancer Hospital, Tongji Medical College, Huazhong University of Science and Technology, Wuhan, China

**Keywords:** cholangiocarcinoma, immunotherapy, radiotherapy, radiation dose-fractionation, tumor microenvironment

## Abstract

Malignant tumors of the biliary tract exhibit a high degree of malignancy and heterogeneity with a poor overall prognosis. Immunotherapy has limited benefits for patients with cholangiocarcinoma. Radiation therapy can change the tumor microenvironment, but its effect heavily depends on radiation dose and fraction. We report a case of advanced intrahepatic cholangiocarcinoma in a 43-year-old male patient, with a huge liver mass of 16.5 cm in diameter, with bone and liver metastases at the first diagnosis. First-line treatment with chemotherapy and PD1 inhibitor was sustained only for 8 months. In second-line treatment, radiotherapy was administered, with 5 Gy in 5 fractions administered to the entire tumor area and 25 Gy in 5 fractions to the solid lesions of the tumor. After the completion of radiotherapy, programmed cell death 1 inhibitor combined with tyrosine kinase inhibitor was maintained. The patient achieved a progression-free-survival time of 12 months and an overall survival time of 25 months. The success of our case suggests that mixed low- and high-dose radiation can significantly improve tumor control and survival time. In clinical practice, based on the characteristics of the tumor and existing treatment options, the rational combination of existing treatment regimens can improve the prognosis of cholangiocarcinoma.

## 1 Introduction

Biliary tract cancer (BTC) is a type of malignant tumor with low incidence but extreme heterogeneity and high malignancy in the digestive system (1). It arises from multiple risk factors such as bile duct disorders, liver diseases, metabolic and endocrine disorders, and so on (2–4). Compared with the West, the incidence rate of BTC in some eastern regions is much higher, with the incidence rate of intrahepatic cholangiocarcinoma increasing year by year (2, 5). Based on anatomical location, biliary tract malignancies can be divided into intrahepatic, extrahepatic, and gallbladder cancer but regardless of the location of the primary tumor, the overall prognosis is extremely poor (1, 5, 6). Monotherapy with chemotherapy for patients with late-stage disease only provides a median survival period of 8-10 months, while combination with immunotherapy can extend the median survival time to 12-18 months, but the overall efficacy is still limited (5, 7, 8). Recent studies have shown that the microenvironment around tumors plays an important role in influencing the efficacy of anti-tumor therapy, and immunosuppressive microenvironments can induce the resistance to existing treatments, especially to immunotherapy. Therefore, recognizing the characteristics of tumor microenvironment and reversing the immunosuppressive microenvironment will effectively achieve the breakthrough of therapeutic effect.

Radiotherapy is an effective local treatment that can induce tumor cell apoptosis and improve tumor control. It can increase the surgical resection rate and improve prognosis in neoadjuvant, adjuvant, and palliative treatment for biliary tract cancer (9). Additionally, radiotherapy can reverse resistance to immunotherapy and enhance the immunotherapeutic effects of drugs such as programmed cell death 1 (PD-1), programmed cell death ligand 1 (PD-L1), and cytotoxic T-lymphocyte associated antigen-4 (CTLA-4) (10). This is due to the ability of effective radiotherapy to alter the tumor microenvironment, such as improving tumor microvascular permeability, reducing hypoxia, increasing the release of inflammatory factors such as tumor necrosis factor-α (TNF-α), interferon-γ (IFN-γ), interleukin (IL), and inducing infiltration by effective immune cells, such as effector T cells, or even inducing macrophage polarization, thereby improving the response to immunotherapy (10–12). However, the effect of radiotherapy on the tumor microenvironment depends on the radiation dose and fraction (10). The appropriate radiation dose and fraction can reverse the immunosuppressive environment and achieve ideal drug sensitization. Otherwise, it may have the opposite effect and even worsen the immunosuppressive microenvironment, inducing immune resistance. Generally, low-dose radiotherapy induces blood vessel normalization and improves hypoxia, while high-dose radiotherapy promotes tumor cell death and induces immune effector cell infiltration (10). Therefore, radiation therapy has a double-edged sword effect on tumor microenvironment (13). Based on the tumor’s imaging and pathological characteristics, selecting appropriate radiation doses and fractions for different individuals will be conducive to enhancing existing treatment efficacy.

The combination of immunotherapy and radiotherapy has been studied for biliary tract cancer, but currently disclosed data have not shown encouraging results. Here, we report a case of a patient with advanced intrahepatic biliary tract cancer who failed standard chemotherapy comminated with immune therapy. We used a targeted combination of PD1 inhibitor and selected different-dose-fraction radiotherapy based on the tumor’s characteristics in imaging and pathology, achieving effective tumor control and survival benefit.

## 2 Case description

**Figure 1:**
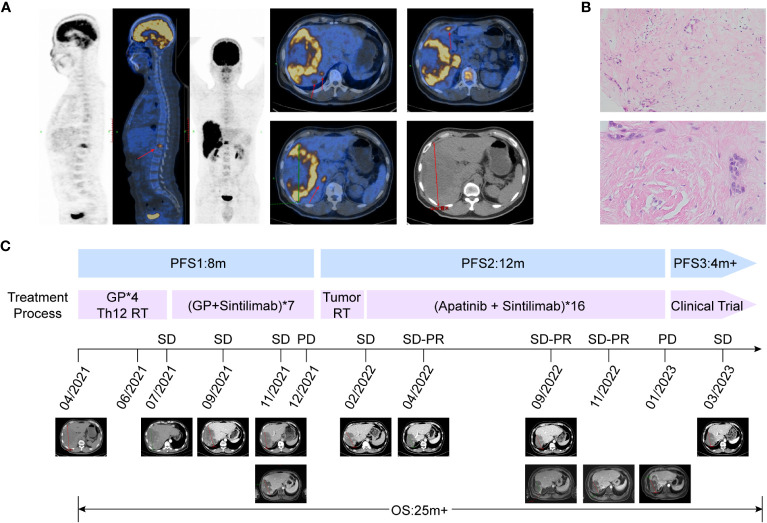
Representative images, pathological features and clinical courses of this patient. **(A)** PET/CT shows that the patient has multiple liver metastases and pyramidal metastases at initial diagnosis, indicating stage IV intrahepatic cholangiocarcinoma. **(B)** Hematoxylin-eosin staining images of the pathological section show a small amount of tumor tissue with the concentrated distribution of active foci, and most of the edges are mucous tissue. **(C)** Patient’s clinical courses and treatment response assessment: The patient entered second-line therapy in January 2022, and achieved SD-PR 3 months after the end of radiotherapy. The best response was SD-PR, and the second-line PFS reached 12 months.

A 43-year-old man with persistent right epigastric pain and back pain was diagnosed with stage IV intrahepatic cholangiocarcinoma in April 2021. Imaging examinations showed a huge liver mass on enhanced CT and multiple slightly low-density lesions in the liver on positron emission tomography-computed tomography (PET-CT), with the largest lesion measuring 16.5×11.6 cm in the right liver lobe and T12 vertebral body destruction (**Figure 1A**). Serum tumor markers displayed the elevated AFP levels with 353 ug/L, and CA125 with 169.9 U/ml. Pathology examination showed a large amount of mucin in the puncture tissue, with a few poorly differentiated adenocarcinoma cells visible (**Figure 1B**). Immunohistochemical staining was positive for PCK, CK7, and CK19, but negative for glypican3, AFP, hepatocyte, P40, CD34, ERG, CD31, S-100, SMA, desmin, and positive for INI-1. Genetic testing did not detect any significant gene mutations, and TMB was 6.82 mutations/Mb. Thus, the patient was diagnosed as intrahepatic cholangiocarcinoma and the disease has achieved stage IV based on the eighth edition AJCC staging of tumor (14). Surgery was not feasible and systemic treatments, such as chemotherapy and/or immunotherapy, were recommended according to current guidelines.

From April to July 2021, the patient first received 4 cycles of chemotherapy of GP regimen (gemcitabine 1000 mg/m^2^, day1 and day8; cisplatin 75 mg/m^2^, day1, every 3 weeks), accompanied with radiotherapy to the 12th thoracic vertebra (**Figure 1C**). Imaging evaluation showed the tumor diameter was reduced from 16.5 to 15.7 cm, and the treatment response of stable disease (SD) was achieved according to Response Evaluation Criteria in Solid Tumors (version 1.1) (**Figure 1C**), while serum AFP and CA125 were slightly decreased. Consideration of the limited efficacy of chemotherapy alone, Sintilimab, a PD-1 inhibitor, was added to the treatment regimen in July 2021(200 mg every 3 weeks). The patient received a combination of the PD-1 inhibitor and GP regimen for 7 cycles from July to December 2021 in further. Subsequent treatment response evaluation still showed SD based on CT image; but further confirmation by magnetic resonance imaging revealed an increase in solid lesions in the tumor, suggesting local disease progression, with PFS for first-line treatment of 8 months (**Figure 1C**).

**Figure 2:**
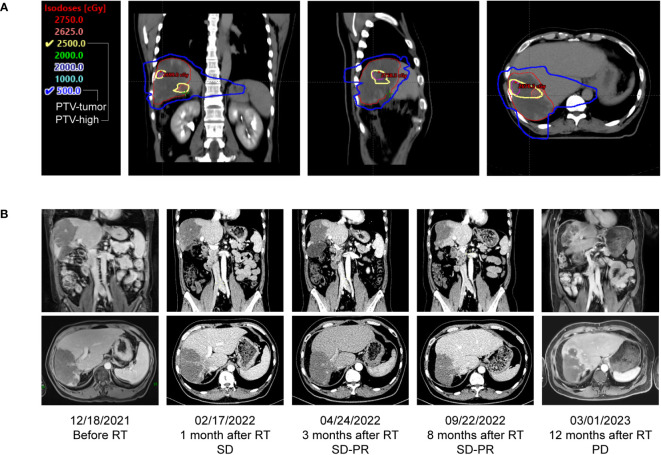
Dose distributions and comparison of treatment response before and after radiotherapy. **(A)** Dose distribution of transverse, coronal and sagittal view. Based on MR images, the radiation treatment plan for the patient’s parenchymal tumor lesion was 25 Gy in 5 fractions, presented as yellow line, and the plan for the overall tumor area was 5 Gy in 5 fractions, presented as blue line. **(B)** Coronary and transverse image changes of the tumor before and after radiotherapy, the best response was PR after radiotherapy.

As the patient refused further chemotherapy, intensity modulated radiation therapy (IMRT) was delivered to the liver lesion area in January 2022. Based on MRI imaging which revealed the massive low-density regions within the tumor area, and only solid cancer foci were identified near the blood vessels at the margin of the tumor (**Figure 1C**). The overall tumor area was declined and defined as gross target volume (GTV-tumor), meanwhile, local high-density solid tumor foci within the overall low-density tumor area were delineated and defined as GTV-high, and the relevant planning target volume (PTV) was formed by 5mm expansion from the margin of corresponding GTV. A low-dose radiation of 5Gy/5F was delivered to PTV-tumor, while, a high-dose radiation of 25Gy/5F was administered to the PTV-high area (**Figure 2A**). After radiation therapy, the patient received further treatment with Sintilimab and tyrosine kinase inhibitor (TKI), Apatinib (500 mg, orally once daily). In April 2022, imaging evaluation showed a significant reduction in the tumor size, with a stable disease close to partial response (SD-PR) achieved (**Figure 2B**), with normalized AFP and CA125 levels. The patient continued with Apatinib and Sintilimab until January 2023, when imaging evaluation showed an increase in the solid lesion at the tumor edge, indicating disease progression. At that time, the PFS of radiation therapy combined with targeted immunotherapy was 12 months, and the patient’s overall survival (OS) reached 25 months from the time of initial diagnosis (**Figure 1C**).

**Figure 3:**
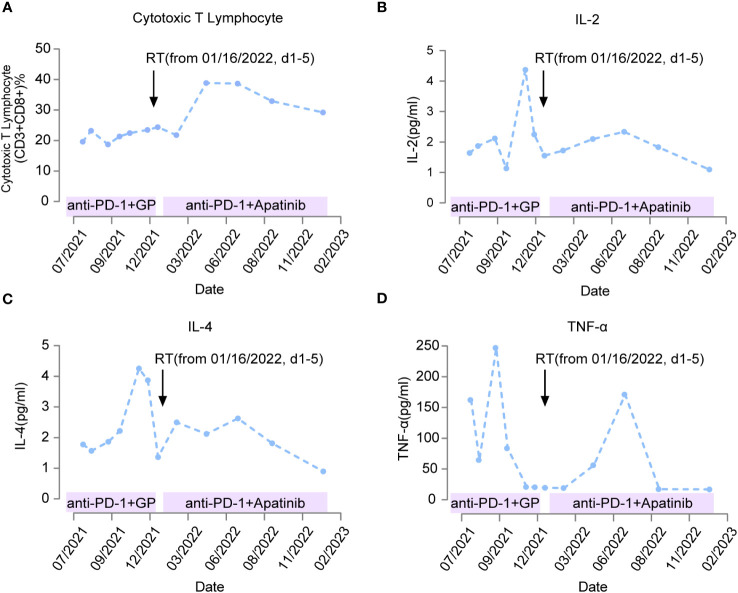
Changes in immuno-related indicators of patient response during treatment. The treatment plans for patients at different time periods are marked on the horizontal axis. Black arrows indicate the time point to radiotherapy (from January 16, 2022, d1-5). When the anti-PD-1 therapy was combined with chemotherapy, the change of killer T lymphocytes with anti-tumor effect was not obvious as shown in **(A)** IL-2/TNF-α/IL-4 rises rapidly and then decreases significantly as shown in **(B–D)** Anti-PD-1 therapy after radiotherapy can significantly increase the level of killer T lymphocytes as shown in A, IL-2/TNF-α/IL-4 was maintained at high levels for a long time from 2 months after radiotherapy as shown in **(B–D)**.

During the course of immunotherapy, we concurrently monitored lymphocytes and inflammatory cytokines (**Figure 3**). We found that the ratio of CD3+ and CD8+ T cells, which are cytotoxic T lymphocytes (CTL), remained at a low-level during immunotherapy combined with chemotherapy, slowly increased after radiation therapy was added, and peaked three months after radiation therapy. It then maintained a high level, which was consistent with the duration of disease control. However, in January 2023, when imaging showed disease progression, the ratio of CD3+ CD8+ T cells also decreased to a low point. This result suggests that radiation therapy can increase the distribution and ratio of CTL in the blood circulation, and is closely related to the synergistic effect of immunotherapy (**Figure 3A**).

In addition to monitoring the distribution of lymphocytes during treatment, we also monitored the change in inflammatory cytokines levels (**Figure 3**). We found that serum levels of IL-2 and IL-4 remained low after starting immunotherapy in July 2021, but increased significantly in November 2021, coinciding with the enlargement of the tumor and clinical evaluation showing disease progression. After radiation therapy was administered in January 2022, serum levels of IL-2 and IL-4 decreased again and remained low for nearly 10 months (**Figures 3B, C**). These results suggest that administration of immunotherapy combined with chemotherapy can suppress the secretion of IL-2 and IL-4; however, the overall suppression time is only 4 months. The increase of IL-2 and IL-4 can be reversed and the level of suppression can be maintained for a longer period after the delivery of radiation therapy. At the same time, we also measured the expression of TNF-α in the serum. We found that TNF-α increased two months after immunotherapy was initiated, and then decreased to a low point after four months of treatment, and its low serum level was synchronous with the imaging showing tumor progression. After radiation therapy combined with TKI and immunotherapy, TNF-α increased again, peaked three months after radiation therapy, and then decreased to a low point after 8 months of treatment. The overall duration of maintaining a high level was longer than the earlier immunotherapy combined with chemotherapy (**Figure 3D**). This suggests that radiation therapy combined with immunotherapy is more favorable for the release of the antitumor inflammatory cytokine TNF-α.

## 3 Discussion

The emergence of immunotherapy has shown breakthrough efficacy in the treatment of various solid tumors (15, 16). Similarly, in the treatment of advanced biliary tract cancer, immunotherapy combined with chemotherapy has enhanced survival benefits compared to that of chemotherapy alone, but the benefits are limited (8). Biliary tract malignant tumors are tumors with extreme temporal and spatial heterogeneity (17, 18). Different anatomical sites, pathological types, and tumor development stages are often accompanied by corresponding changes in the tumor microenvironment (17). Existing studies have shown that tumors with significant immune cell infiltration in the tumor microenvironment tend to exhibit enhanced sensitivity to immunotherapy, while tumors with low immune infiltration and more stromal cell infiltration tend to exhibit immune resistance. Therefore, based on the degree of immune cell and stromal cells infiltration in the microenvironment, biliary tract malignant tumors are also classified into “hot” and “cold” immunotype (17–19). Thus, the characteristic of the tumor microenvironment is an important factor affecting the efficacy of immunotherapy.

For advanced biliary tract malignancies, based on existing clinical data, the median PFS obtained by single targeted combined immunotherapy in second-line treatment is only in 5–6 months, and the survival benefit is limited (8). In our case, both PFS and OS are shown to exceed the results of existing studies. The main characteristic of this case is that both pathological and imaging findings revealed poorly vascularized, extensive necrosis and mucinous components, indicating an immunologically “cold” tumor phenotype. Therefore, the initial chemotherapy and immunotherapy were not effective, suggesting that changing the tissue microenvironment would be beneficial for enhancing immunotherapy efficacy. Existing studies have shown that the tumor microenvironment is composed of tumor blood vessels, tumor-associated immune cells, and stroma, forming the corresponding tumor vascular microenvironment, tumor immune microenvironment, and tumor matrix environment. These three components complement each other to promote tumor growth, invasion, and resistance to treatment (13, 20, 21). The blood vessels in tumor tissue, also known as tumor neovascularization, have structural and functional defects compared to normal blood vessels and are prone to induce tissue hypoxia (22, 23). This leads to the massive release of inflammatory factors such as vascular endothelial growth factor (VEGF), colony stimulating factor 1 (CSF1), IL-2, and IL-4, which attract immunosuppressive cells such as tumor-associated macrophages, tumor-associated neutrophils, myeloid-derived suppressor cells (MDSCs), and tumor-associated fibroblasts to infiltrate and accumulate around the tumor, further inducing the release of negative inflammatory factors, collectively promoting an immunosuppressive microenvironment and leading to resistance to immunotherapy (13, 22–24). Therefore, a clear identification of immunotypes of tumor microenvironment based on existing diagnostic methods such as pathology, imaging, lymphocyte populations, and inflammatory factor analysis is necessary for the selection of appropriate anti-tumor treatment regimen.

Radiation therapy has been found to affect changing the structure and function of the tumor microenvironment, but its impact depends on the radiation dose and fractionation (10, 21). Single-dose and single-dose-fraction radiation therapy cannot provide the same immunological effects for different types of tumors (10). Existing radiation therapy modes are divided into conventional radiotherapy (1.8-2 Gy), low-dose radiotherapy (0.1-1 Gy), and high-dose radiotherapy (>5 Gy) based on the single fraction dose (10, 25). Conventional radiotherapy is a more commonly used radiation therapy mode, but conventional single-dose (1.8-2 Gy) radiation therapy is prone to clearing lymphocytes in the irradiated area, reducing the aggregation of tumor-specific lymphocytes, and exacerbating the negative effects of immune suppression (10, 25). Low-dose radiation therapy can induce tumor apoptosis but cannot achieve rapid induction of tumor necrosis. However, low-dose radiation therapy can induce normalization of abnormal tumor vessels, increase tumor tissue oxygenation, and improve hypoxic conditions (13, 26). It can also attract effector T cells, such as CD4+ and CD8+, to aggregate in the tumor area, thereby improving the vascular microenvironment and immune microenvironment of the tumor area (13, 27). High-dose radiation therapy can induce tumor necrosis through a single large dose of irradiation rather than the apoptotic effect observed with low-dose irradiation. Therefore, it can effectively promote the release and presentation of tumor antigens, while increasing the release of inflammatory factors such as IFN-γ and TNF-α in tumor tissue. This can reduce the infiltration of suppressive immune cells such as regulatory T cells (Tregs) and M2-type macrophages (13, 28). Therefore, generally, different doses of radiation therapy exert their advantages in regulating the tumor microenvironment status. Among them, low-dose radiation has advantages in improving tumor vascular status and tissue hypoxia, while high-dose radiation therapy can induce tumor necrosis, release of tumor antigens, and promote the emergence of effective antitumor immune responses. As Savage et al. found, radiation therapy with 0.5 Gy × 4 fractions can significantly reduce the risk of tumor metastasis (29). A combination of high and low-dose irradiation can significantly improve the locoregional control of tumors and effectively improve survival.

In this case, it is important to consider the dose and fractionation scheme as well as the tolerance of the organs at risk in the design of the radiation therapy plan. The characteristic of this case is that the tumor volume is large, and the residual liver tissue capacity is limited. Previous radiation therapy was given to the 12th thoracic vertebrae, and if high-dose radiation therapy is implemented for the overall liver lesion, there is a possibility of liver damage and adjacent spinal cord overdose, which is not a favorable way to improve the tumor microenvironment. Therefore, we adopted low-dose radiation therapy for the entire tumor with a single dose of 1 Gy×5 fractions and 5 Gy×5 fractions radiation therapy for the solid lesion in the tumor. Three months after radiation therapy, blood lymphocyte, and inflammatory factor analysis showed a significant increase in the CTL as CD3+CD8+ cells ratio, accompanied by an increase in the inflammatory factors TNF-α and a decrease in IL-2 and IL-4, indicating an increase in immune activity. Meanwhile, imaging revealed significant tumor regression and subsequent follow-up showed that the PFS after radiotherapy reached 12 months. The results suggested that a mixed dose of radiation therapy is more advantageous for improving the tumor microenvironment and stimulating antitumor immune response, and the effect time may be around three months after radiation therapy. Moreover, adding immunotherapy combined with TKI after radiation therapy can effectively induce tumor remission, indicating that radiation therapy before immunotherapy may be a more effective treatment sensitization strategy.

In summary, biliary tract malignant tumors are highly heterogeneous in the field of malignancies, not only in terms of tumor genetic heterogeneity but also in terms of microenvironment heterogeneity. Therefore, in the design of treatment plans for late-stage patients, it is necessary to base them on the characteristics of the tumor and consider the characteristics of the tumor tissue microenvironment. This involves the rational planning and application of existing treatment methods, including chemotherapy, targeted therapy, immunotherapy, and radiation therapy, thereby overcoming treatment resistance and achieving enhanced survival benefits.

## Data availability statement

The original contributions presented in the study are included in the article/supplementary material. Further inquiries can be directed to the corresponding author.

## Ethics statement

Written informed consent was obtained from the individual(s) for the publication of any potentially identifiable images or data included in this article. Written informed consent was obtained from the participant/patient(s) for the publication of this case report.

## Author contributions

NW: Data curation, Writing – review & editing. AH: Writing – original draft. LW: Investigation, Writing – original draft. JH: Software, Writing – review & editing. TC: Software, Writing – original draft. XM: Data curation, Writing – review & editing. JY: Funding acquisition, Resources, Writing – original draft. YX: Funding acquisition, Writing – original draft. HM: Data curation, Funding acquisition, Methodology, Resources, Writing – original draft.
